# When did coronavirus arrive in Europe?

**DOI:** 10.1007/s10260-021-00568-4

**Published:** 2021-05-20

**Authors:** Augusto Cerqua, Roberta Di Stefano

**Affiliations:** 1 Department of Social Sciences and Economics, Sapienza University of Rome, P.le Aldo Moro, 5, 00185 Rome, Italy; 2 Department of Statistical Sciences, Sapienza University of Rome, Viale Regina Elena 295, 00161 Rome, Italy

**Keywords:** Coronavirus, Europe, Counterfactual approach

## Abstract

The first cluster of coronavirus cases in Europe was officially detected on 21st February 2020 in Northern Italy, even if recent evidence showed sporadic first cases in Europe since the end of 2019. In this study, we have tested the presence of coronavirus in Italy and, even more importantly, we have assessed whether the virus had already spread sooner than 21st February. We use a counterfactual approach and certified daily data on the number of deaths (deaths from any cause, not only related to coronavirus) at the municipality level. Our estimates confirm that coronavirus began spreading in Northern Italy in mid-January.

## Introduction

Recent evidence shows sporadic first cases of coronavirus (SARS-CoV-2) in Europe since the end of 2019. For instance, it has been confirmed that a patient hospitalized on 27th December for suspected pneumonia near Paris had coronavirus as claimed in France 24 (2020). Likewise, a German businessman with mild symptoms tested positive with coronavirus on the 27th January 2020 (Rothe et al. 2020). The first official case in Italy was detected on 21st February 2020 in the Northern area. Moreover, there is increasing anecdotal evidence that the virus might have reached Italy sooner, with a consequent early spread leading to the pandemic expolosion in late February. According to a survey conducted by the television broadcast Report, aired on 30th March, it seems that there had already been a large number of pneumonia cases at the start of 2020 in Northern Italy, particularly in Piacenza (Emilia-Romagna), located 18 km from Codogno (Lombardy) where the first Italian case of coronavirus was officially reported. A press review (LIBERTÀ 2020; CORRIERE DELLA SERA 2020b; CORRIERE DELLA SERA 2020a) identifies that on 30th December, the Piacenza Hospital had 40 cases of pneumonia in the previous week, and on 7th January, Milan had a peak of pneumonia cases with requests for extra hospital beds. These pneumonia cases had similar characteristics to interstitial pneumonia caused by coronavirus, even if no tests were done to confirm the virus that caused them. Medical professionals did not attribute these cases to coronavirus. The medical protocols to test for the presence of the virus involved not only that the patient had respiratory problems, but also that he/she had come from China, or that he/she was in contact with people coming from China. This means that as those infected spread the disease, everybody was looking for patient zero, i.e., the patient coming from China, but nobody was looking at patient one, i.e., the patient not directly connected with China. The possibility that the virus might have spread in Italy long before 21st February is quite likely also considering that from 17th November, i.e., the date of the first case in Wuhan (China) to 31st January, i.e., the date in which Italy suspended flights to and from China, there were 203,894 arrivals from China, of which 15,400 from Wuhan to Fiumicino (Rome) and 125,000 to Malpensa (Milan). Moreover, by analyzing the first 5,830 laboratory-confirmed cases in Lombardy through standardized interviews of confirmed cases and their close contacts, Cereda et al. (2020) estimate that the virus reached Southern-Lombardy around a week before the case of Codogno.

This paper aims at assessing the plausibility that coronavirus spread in Italy before 21st February and to investigate approximately when. In light of anecdotal evidence, in the main analysis, we focus on Piacenza as a case study, while we report the analysis on other municipalities in the Appendix. We analyze official daily data on the number of deaths made available by the Italian National Institute of Statistics (Istat)^1^ for 7,904 municipalities for the period 1st January - 31st August 2020^2^. Therefore, we need to compare Piacenza with a scenario where the virus did not hit until 21st February 2020. To this aim, we must estimate a valid counterfactual scenario using as control group municipalities with similar characteristics to Piacenza, but which are less likely to have been affected by the virus before 21st February 2020. Counterfactual approaches are usually adopted to estimate the impact of a specific policy change on an outcome of interest. Although many scholars used counterfactual approaches to investigate research questions linked to COVID-19 (see, among others, Bayat et al. (2020), Cole et al. (2020), Mitze et al. (2020))^3^, to our knowledge, no study used counterfactual methods to estimate when coronavirus spread in Europe. In this paper, we make unconventional use of this evaluation approach as we consider as policy change the possible diffusion of coronavirus in Piacenza earlier than 21st February 2020. The method adopted is the trajectory balancing method, recently developed by Hazlett and Xu (2018). As the potential date for the beginning of the coronavirus in Italy, we use 21st January. This choice is a trade-off between available data and having at least 20 pre-treatment time periods to estimate the counterfactual scenario.

The paper is organized as follows. In the next section, we present the identification strategy, while Section 3 describes the data. The following section shows our empirical findings, including a placebo test and several robustness checks. Section 5 concludes and discusses policy implications.

## Methodology

To assess if coronavirus was present in Piacenza earlier than 21st February 2020, we adopt a novel counterfactual approach, the trajectory balancing method (TB) developed by Hazlett and Xu (2018). TB is a general reweighting approach for causal inference, which builds upon the synthetic control method (SCM), developed by Abadie and Gardeazabal (2003) and Abadie et al. (2010), enabling to estimate the treatment effect in the presence of one or a few treated units. The idea is to compare the cumulative daily number of deaths per 10,000 inhabitants (the outcome of interest) observed in Piacenza (the treated unit) before the 21st February with a ‘synthetic’ Piacenza, that represents what would have happened to the cumulative daily number of deaths per 10,000 inhabitants in Piacenza if there was not coronavirus. If the former substantially exceeds the latter, TB suggests that there were ‘unexpected deaths’ (the treatment effect), i.e., that coronavirus (the treatment) was already widespread in Piacenza before the 21st February. In a difference-in-differences (DiD) setting, TB allows us to construct, transparently, the ‘synthetic’ Piacenza in the absence of treatment. We use TB in an unusual way as we do not know the exact treatment date, i.e., when coronavirus arrived. Determining such date is our primary goal. To this end, we have selected 21st January 2020, just a month before the official day, as the potential treatment date. In this way, the 20 days from 1st January to 20th January represent the pre-treatment period and help us build the ‘synthetic’ of Piacenza. Whilst, days from 21st January to the 21st February represent the post-treatment, i.e., the period where we can observe the ‘unexpected’ deaths. The ‘synthetic’ of Piacenza is given by a weighted average of control units (municipalities that we consider as not affected by coronavirus) whose pre-treatment characteristics closely match those observed in Piacenza. We then consider the difference between the trend of cumulative deaths observed in Piacenza and the trend of ‘synthetic’ Piacenza to determine whether coronavirus was already spread in Piacenza before 21st February. More specifically, if the cumulative death trend of Piacenza moves away from the counterfactual estimate before 21st February 2020, we might argue that there were people infected by coronavirus before this official date.

In the main analysis, we use a panel dataset of 33 Italian municipalities (Piacenza and other 32 municipalities with similar characteristics to Piacenza), observed for the period 1st January 2020–21st February 2020. Building the ‘synthetic’ Piacenza implies choosing weights for each of the other municipalities *i* such that the weighted average of cumulative deaths and other characteristics (described in Section 3) are approximately equal in the pre-treatment period to Piacenza. The TB chooses a set of non-negative weights *w* such that their sum is equal to one. TB employs a balancing procedure on the P principal components of the pre-treatment matrix that includes the outcome variable and other pre-treatment characteristics. Thus, the unexpected cumulative deaths in Piacenza $\theta _{t}$ in each post-treatment period ($t > 20th \ January$) is given by the difference between the cumulative deaths observed for Piacenza and those observed for the ‘synthetic’ Piacenza, as follows:

1 $$\begin{aligned} \theta _{Piacenza,t}=Y_{Piacenza,t}-\sum _{i=1}^{32}w_iY_{i,t} \ \ \ \ \ \ \ \ \ \ for \ \ \ \ t>20th\ January \end{aligned}$$

where $Y_{it}$ is the cumulative daily number of deaths per 10,000 inhabitants observed for a generic municipality *i* that belongs to the donor pool after 20th January and $w_i$ is the synthetic control weight. Besides, TB never directly fits a model; hence, the possibility of an erroneous extrapolation based on estimated model parameters is minimized. We employ the tjbal command in R developed by Hazlett and Xu (tjbal package for R is available at https://yiqingxu.org/software/tjbal/tjbal.html).

## Data

With the spread of the coronavirus pandemic, an increase in the number of deaths was observed, higher than that officially attributed to coronavirus [see Report Istat-ISS (2020) on the impact of the Covid-19 epidemy on total resident population mortality for the first quarter 2020 for the estimation at the provincial level and Buonanno et al. (2020) for the estimation in Lombardy municipalities]^4^. Monitoring the progress of deaths as a whole, regardless of the cause, is therefore of great interest (see Magnani et al. 2020, for a descriptive study). Istat released data on the daily number of deaths (deaths from any cause, not only related to coronavirus) for all 7,904 Italian municipalities. Considering the evidence coming from the survey conducted by the television broadcast Report, we consider the municipality of Piacenza as the treated unit to verify if the virus was present before 21st February. Our research does not compare the number of deaths in 2020 with the average number of deaths of previous years^5^ nor do we compare the observed number of deaths with the time series (expected value), as in the SISMG^6^ report. On the contrary, we adopt the counterfactual approach TB, with the idea that a linear combination of units not affected by the intervention could represent what would have happened to the treated unit better than the aforementioned approaches. Like time-series modeling, TB takes into account unobserved factors (for example, flu epidemics), which can also vary over time. To construct the ‘synthetic’ unit of Piacenza, we limit the set of potential control units, commonly named donor pool, to the 32 municipalities located in the North of Italy^7^ with a population size similar to Piacenza (between 50% more and 50% less of the Piacenza population). As suggested in Abadie et al. (2015), by restricting the donor pool to municipalities with characteristics more similar to Piacenza, we reduce the risk of interpolation bias. The municipalities in the same geographical area have similar local economic structures and sector specialization, factors that can act as a vehicle of disease transmission (see Ascani et al. 2020 for details). In other words, we consider the municipalities in which the virus could spread equally^8^. Moreover, the same geographical area means a similar impact of seasonal risk factors (climatic conditions and flu epidemics). To build a ‘synthetic’ unit as close as possible to Piacenza, we use the following predictors: the average number of deaths in the first 20 days of the years 2015-2019, the total number of deaths in 2019, the total population recorded on the 1st January 2020, the share of the population aged over 65, the number of employees in 2018, and the proportion of those employed in manufacturing^9^. TB builds the ‘synthetic’ Piacenza as a weighted average of Monza, Cremona, Rimini, Cinisello Balsamo, Trento, Gallarate, Treviso, and Udine, as described in Table 3 in the Appendix^10^.

## Results

**Fig. 1:**
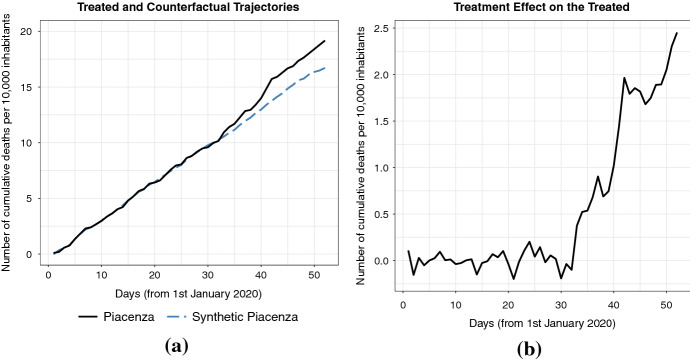
Trends and gap in the daily number of cumulative deaths per 10,000 inhabitants: Piacenza and synthetic Piacenza

Panel (a) of Figure 1 shows the trends in the daily number of cumulative deaths per 10,000 inhabitants since 1st January of the municipality of Piacenza (dark line) and the ‘synthetic’ Piacenza (dashed line), i.e., the weighted outcome of the 32 municipalities based on the TB approach. The horizontal axis represents the day from 1st January to 21st February, while the vertical axis represents the number of deaths per 10,000 inhabitants. As previously explained, we consider 21st January to be the possible date of the beginning of contagion. The figure shows that the death trend follows its synthetic counterpart very closely pre-treatment as well as until the end of January. From the beginning of February, we observe an increasingly positive gap between the trends, which on 21st February amounts to +2.45 more deaths per 10,000 inhabitants. This means that in Piacenza, a municipality with 104,000 inhabitants, we observe approximately 25 deaths more than predicted by the counterfactual scenario. Since the beginning of February, the detected ‘unexpected’ deaths imply that the virus was already spread for some time in Piacenza. The gap, i.e., the difference between Piacenza and its ‘synthetic’ counterpart, is presented in Panel (b) of Figure 1. The extremely good fit between Piacenza and its ‘synthetic’ version in the absence of coronavirus is also confirmed in Table 1 that reports the covariates balance in the pre-treatment period, and the average of the 32 municipalities in the donor pool.

**Table 1:** Covariate balancing

	Piacenza	Controls mean	Synthetic piacenza (TB)
Share of 65+ population (1st Jan 2020)	0.244	0.252	0.247
Total population (1st Jan 2020)	104,315	87,383	106,107
Total deaths (2019)	1,215	1,010	1,133
Avg deaths in the first 20 days (2015-19)	3.900	3.425	3.795
Share of empl. in manufacturing (2018)	0.139	0.164	0.144
Total employees (2018)	43,900	32,395	39,443

### Placebo test

**Fig. 2:**
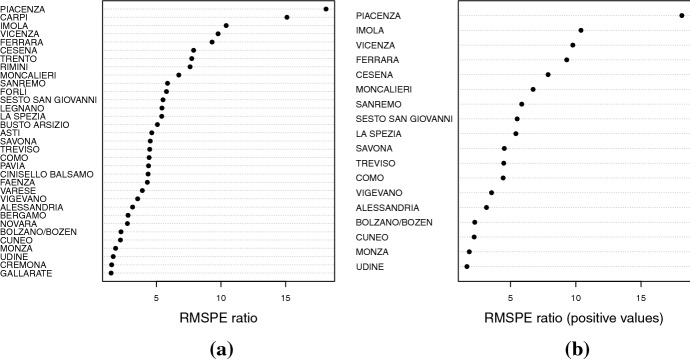
Ratio of Post-21st January 2020 RMSPE to Pre-21st January 2020 RMSPE: Piacenza and control municipalities

To evaluate the significance of the results, we run an in-space placebo test, i.e., we reassign the treatment to each of the 32 municipalities that make up the ‘synthetic’ Piacenza, where we presume that the coronavirus arrived later. We will deem the effect of the arrival of the virus in Piacenza statistically significant if the estimated effect is large relative to the distribution of placebo effects. We follow Abadie et al. (2015) and show in Panel (a) of Figure 2 the ratios between the post-21st January Root Mean Square Prediction Error (RMSPE) and the pre-21st January RMSPE for Piacenza and all 32 municipalities. RMSPE measures the magnitude of the gap in the outcome variable between each municipality and its ‘synthetic’. A large gap between the post- and pre-presumed date of the first contagion indicates a relevant effect, i.e., an unusual pattern of deaths compared to the counterfactual counterpart. As shown in Panel(a) of Figure 2, Piacenza stands out as the municipality with the highest RMSPE ratio. As this test does not take into account whether the placebo unit shows more or fewer deaths than its counterfactual, we repeat the test only on the municipalities with a number of deaths higher than the counterfactual prediction on the date of 21st February. As reported in Panel (b) of Figure 2, we observe that Piacenza ranks first by an even larger margin, confirming the statistical significance of the estimate.

### Robustness checks

As suggested in Abadie (forthcoming), we report in Table 2 the estimates of several robustness exercises, which help us verify the sensitivity of our results to changes in the design of the evaluation approach. Notably, we change:

1. the treatment date, backdating and postdating the treatment by 5 days;
2. the donor pool, considering all municipalities with at least 10,000 inhabitants in the North of Italy, all regions, and municipalities with at least 10,000 inhabitants in all Italian regions. Besides this, we propose a leave-one-out analysis, i.e., we re-run the trajectory balancing, excluding from the sample one-at-a-time each of the municipalities that contribute to the counterfactual^11^;
3. the predictors of the outcome variable, adding a measure of air quality (PM-10) in 2018^12^ and the share of males;
4. the algorithm to assess weights, using the SCM^13^(see Abadie et al. 2010, and Abadie et al. 2015 for more details).

**Fig. 3:**
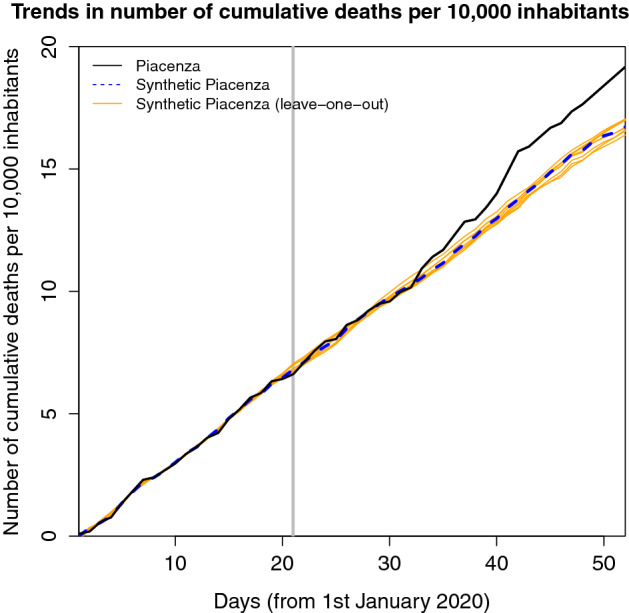
Leave-one-out distribution of the synthetic Piacenza

All robustness checks lead to estimates which are very close to those reported in the main analysis. Moreover, in Figure 3, we can observe that the estimates coming from leave-one-out distribution are centered around the synthetic Piacenza, showing that our findings are not driven by the specific weight given to a municipality in the donor pool.

**Table 2:** Robustness checks

Main estimate	Gap in the number of cumulative deaths per 10,000 inhabitants on 21st February
	2.45
*Alternative treatment date*	
Backdating 5 days earlier	2.18
Postdating 5 days later	2.03
*Alternative population thresholds*	
Municipalities with at least 10,000 inhabitants	2.82
Considering all regions	2.45
No population and regional restriction	2.23
Leave-one-out procedure	2.42
*Addition of covariates*	
Adding the quality of air	2.70
Adding the share of males	2.30
*Alternative balancing method*	
SCM	2.99

In the leave-one-out procedure, we consider the average number of cumulative deaths per 10,000 inhabitants on 21st February for the eight iterations

## Conclusion

Our research aims to analyze not only the presence of coronavirus in Italy sooner than 21st February but, more importantly, to assess whether the virus had already spread in a specific territory. As Italy has been the first European country severely hit by the pandemic, we argue that it must be the first European country where COVID-19 spread, except for a few sporadic cases detected throughout the continent since the end of 2019. To test our hypothesis, we adopt the trajectory balancing approach to analyze certified data on the number of deaths at the municipality level. This allows us to avoid underreporting, which seems to be widespread with official data on coronavirus. We find that Piacenza experienced an unexpected increase in the number of deaths since the beginning of February with respect to the counterfactual. This means that coronavirus had already spread in a specific area of Northern Italy since mid-January (considering the delay between contagion and death) and that by 21st February hundreds of individuals were already infected. This finding might help in the historical investigation of how the virus spread in Italy first and then in Europe. Besides, in the era of big data with the spread of digital health, our evidence underlines the need to invest more in data collection systems’ efficiency and timeliness. An effective information system allows us to spot anomalies in the data and helps policymakers handle emergencies by providing a more precise picture of the situation. Moreover, as highlighted by Birrell et al. (2020), in a pandemic context, real-time monitoring is vital to avoid making public health decisions based on misspecified models. The coronavirus emergency has demonstrated that most developed countries lag behind in this technological challenge, calling for prompt and large investments in this sector. For instance, the Italian daily mortality monitoring system collects daily deaths for individuals aged 65 years and older only for the 34 largest municipalities. Higher coverage of the Italian territory and a warning system based on mortality as well as on hospitalization or ICU accesses, might have allowed detecting the presence of coronavirus in Italy a few weeks in advance.

## Figures and Tables

**Fig. 4:**
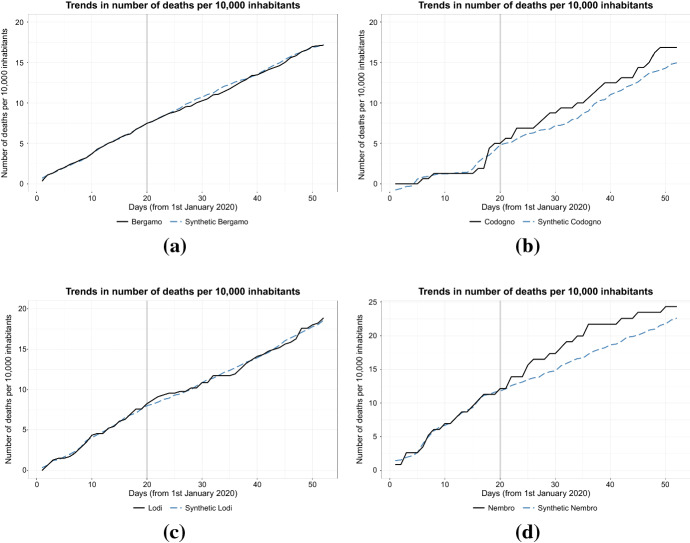
Trends in number of cumulative deaths per 10,000 inhabitants in Bergamo (121,781 inhabitants), Codogno (15,994 inhabitants), Lodi (46,050 inhabitants) and Nembro (11,514 inhabitants)

## Trajectory balancing and Synthetic weights

See Table 3.

**Table 3:** Description of control group

Municipality	Region	Population	TB weights	SCM weights
Piacenza	Emilia-Romagna	104,315	NA	NA
Alessandria	Piedmont	93,634	0	0
Asti	Piedmont	75,528	0	0
Bergamo	Lombardy	121,781	0	0.01
Bolzano	Trentino-Alto Adige	107,407	0	0
Busto Arsizio	Lombardy	83,909	0	0
Carpi	Emilia-Romagna	72,627	0	0
Cesena	Emilia-Romagna	97,190	0	0
Cinisello Balsamo	Lombardy	76,264	0.10	0
Como	Lombardy	85,915	0	0
Cremona	Lombardy	72,672	0.22	0
Cuneo	Piedmont	56,203	0	0
Faenza	Emilia-Romagna	58,953	0	0
Ferrara	Emilia-Romagna	132,195	0	0
Forlì	Emilia-Romagna	118,000	0	0.17
Gallarate	Lombardy	53,935	0.06	0
Imola	Emilia-Romagna	70,000	0	0
La Spezia	Liguria	93,288	0	0
Legnano	Lombardy	60,336	0	0.10
Moncalieri	Piedmont	57,465	0	0
Monza	Lombardy	124,051	0.26	0.21
Novara	Piedmont	103,985	0	0.01
Pavia	Lombardy	73,334	0	0
Rimini	Emilia-Romagna	151,200	0.19	0.10
Sanremo	Liguria	54,850	0	0
Savona	Liguria	59,933	0	0
Sesto San Giovanni	Lombardy	81,841	0	0
Trento	Trentino-Alto Adige	118,902	0.09	0.24
Treviso	Veneto	85,760	0.05	0
Udine	Friuli-Venezia Giulia	99,051	0.02	0.14
Varese	Lombardy	80,645	0	0
Vicenza	Lombardy	111,764	0	0
Vigevano	Lombardy	63,623	0	0

## Other municipalities

Coronavirus hit hard many municipalities in Northern Italy. Some of them possibly had signs of coronavirus before 21st February, as also suggested by Piccininni et al. (2020). In this paragraph, we look at Bergamo, Codogno, Lodi, and Nembro^14^. For each of these municipalities, we use the same model specification and the same criterion for selecting the units to include in the donor pool, i.e., between 50% more or less of the treated municipality population. The results are reported in Figure 4. There is no evidence of a positive gap between the treated and counterfactual trends for Bergamo (Panel (a)) and Lodi (Panel (c)). Conversely, when looking at Codogno (Panel (b)) and Nembro (Panel (d)), there seems to have been an increase in the number of deaths per 10,000 inhabitants from late January. However, considering the small size of these municipalities and the small number of deaths, additional evidence is needed to confirm the potential presence of coronavirus from the end of January.

## Weights leave-one-out

See Table 4.

**Table 4:** Weights Leave-one-out

	Cinisello Balsamo	Cremona	Gallarate	Monza	Rimini	Trento	Treviso	Udine
Alessandria	0.00	0.00	0.00	0.00	0.03	0.05	0.01	0.04
Asti	0.00	0.00	0.00	0.00	0.00	0.00	0.00	0.00
Bergamo	0.00	0.00	0.00	0.00	0.06	0.04	0.00	0.03
Bolzano/Bozen	0.00	0.00	0.00	0.00	0.02	0.01	0.00	0.01
Busto Arsizio	0.00	0.00	0.00	0.00	0.01	0.01	0.00	0.02
Carpi	0.08	0.12	0.00	0.10	0.06	0.09	0.01	0.08
Cesena	0.00	0.00	0.00	0.00	0.00	0.00	0.00	0.00
Cinisello Balsamo		0.00	0.12	0.03	0.01	0.01	0.09	0.01
Como	0.00	0.12	0.00	0.00	0.01	0.03	0.04	0.03
Cremona	0.22		0.24	0.20	0.01	0.01	0.18	0.01
Cuneo	0.00	0.00	0.07	0.02	0.01	0.02	0.00	0.01
Faenza	0.00	0.02	0.00	0.00	0.01	0.01	0.00	0.01
Ferrara	0.00	0.00	0.00	0.00	0.09	0.03	0.00	0.05
Forlì	0.00	0.00	0.00	0.00	0.18	0.07	0.00	0.09
Gallarate	0.05	0.18		0.05	0.01	0.02	0.11	0.01
Imola	0.00	0.00	0.00	0.00	0.02	0.02	0.00	0.01
La Spezia	0.00	0.00	0.00	0.00	0.01	0.01	0.00	0.01
Legnano	0.03	0.00	0.00	0.00	0.00	0.01	0.00	0.00
Moncalieri	0.00	0.00	0.00	0.00	0.00	0.00	0.00	0.00
Monza	0.10	0.00	0.19		0.11	0.11	0.22	0.09
Novara	0.00	0.02	0.00	0.00	0.05	0.05	0.01	0.05
Pavia	0.00	0.00	0.00	0.00	0.00	0.00	0.00	0.00
Rimini	0.23	0.35	0.26	0.28		0.23	0.24	0.20
Sanremo	0.00	0.00	0.00	0.00	0.01	0.02	0.00	0.02
Savona	0.00	0.00	0.00	0.00	0.00	0.00	0.00	0.00
Sesto San Giovanni	0.00	0.00	0.00	0.00	0.00	0.01	0.00	0.00
Trento	0.12	0.00	0.10	0.14	0.12		0.05	0.07
Treviso	0.07	0.00	0.01	0.03	0.01	0.02		0.02
Udine	0.10	0.15	0.02	0.13	0.00	0.01	0.04	
Varese	0.00	0.04	0.00	0.00	0.00	0.01	0.00	0.00
Vicenza	0.00	0.00	0.00	0.00	0.14	0.12	0.00	0.09
Vigevano	0.00	0.00	0.00	0.00	0.01	0.02	0.00	0.02

Column names indicate the municipalities excluded in those iterations

## Footnotes

1 The mortality data recorded by the individual municipalities are acquired within the information system managed by the Ministry of the Interior and then transmitted to Istat, which processes and validates the data.

2 Although granular data on hospitalization or ICU accesses would have been more appropriate to identify the beginning of the spread of the coronavirus epidemic, they are not available.

3 Bayat et al. (2020) analyze the impact of lockdown measures; Cole et al. (2020) estimate the impact of the Wuhan COVID-19 Lockdown on Air Pollution and Health combining machine learning with the augmented synthetic control method, and Mitze et al. (2020) study the effect of face masks on the spread of COVID-19 in Germany.

4 Besides, the official data noise is large and pervasive, especially at the regional and provincial levels (see Peracchi and Terlizzese 2020).

5 In this case, as claimed in a note by Istat, we would observe a reduction in the number of deaths. The phenomenon could be attributed to the reduced impact of seasonal risk factors (climatic conditions and flu epidemics) in the first two months of the year.

6 SISMG is the Italian daily mortality monitoring system that aims to monitor in real-time the daily number of deaths in the elderly population (age 65 years and over) in the 34 biggest Italian municipalities that represent around 20% of the entire Italian population. The expected value is defined as the average per day of the week and number of the week calculated in the five previous years and weighed for the resident population (Istat data) to take into account the progressive aging of the population.

7 Northern regions are: Aosta Valley, Emilia-Romagna, Friuli-Venezia Giulia, Liguria, Lombardy, Piedmont, Trentino-Alto Adige, and Veneto.

8 We are assuming that the municipalities included in the donor pool were not affected by the virus before 21st February 2020. We cannot test such an assumption but, even its failure would only imply, if anything, that our estimates are a lower bound of the true effect.

9 We control for employment as it is likely related to the speed of the spread of the contagion (see Ascani et al. 2020), while the share of employment in manufacturing is a proxy that takes into account that the most vulnerable people are those affected by respiratory diseases that are more widespread in industrialized areas. Data come from the Statistical Register of Active Enterprises (ASIA) archive. ASIA is produced by Istat and covers the universe of firms and employees of industry and services.

10 The Appendix reports the analysis on other municipalities that, to our knowledge, were potentially affected by coronavirus before 21st February. Moreover, in a robustness check, we remove one-at-a-time the municipalities that have positive weights in the building of ‘synthetic’ Piacenza, and the results are unchanged.

11 Table 4 in the Appendix reports the TB weights assigned for each iteration of the leave-one-out procedure.

12 PM-10 is an air pollutant that could serve as a carrier for viruses. Setti et al. (2020) highlighted the relationship between the rapid COVID-19 infection spread in Northern Italy and PM-10 pollution. Air pollution data is from 573 monitoring stations distributed across the Italian territory. We employ the kriging spatial interpolation to impute the PM-10 average yearly value for each municipality. Given the likely measurement error of this variable, we control for it only in this robustness check.

13 The SCM weights are shown in Table 3 in the Appendix.
